# Periapical Lesion Healing After Retreatment and Root Canal Filling with a Bioceramic-Based Sealer: A Randomized Clinical Study with 1-Year Follow-Up

**DOI:** 10.3390/jcm14238267

**Published:** 2025-11-21

**Authors:** Boris Pažin, Tomislav Lauc, Gabrijela Kapetanović Petričević, Dragana Gabrić, Ivona Bago

**Affiliations:** 1 Department of Endodontics, Oral Patology and Periodontology, Dental Polyclinic Zagreb, 10000 Zagreb, Croatia; bpazzin@gmail.com; 2 Dental Polyclinc Apolonija, 10000 Zagreb, Croatia; tomislav.lauc@gmail.com; 3 Department of Endodontics and Restorative Dentistry, School of Dental Medicine, University of Zagreb, 10000 Zagreb, Croatia; gkapetanovic@sfzg.hr; 4 Department of Oral Surgery, School of Dental Medicine, University of Zagreb, 10000 Zagreb, Croatia; dgabric@sfzg.hr

**Keywords:** calcium silicate-based sealers, endodontic outcome, single-cone technique, cone beam computed tomography

## Abstract

**Background/Objectives:** The clinical outcome of root canal treatments using calcium silicate-based sealer (CSBS) remains unclear. This study aimed to evaluate the 1-year effect of CSBS in combination with a single-cone obturation technique on periapical lesion (PL) healing evaluated on cone beam computed tomography scans (CBCT) after single-visit root canal retreatment. **Methods**: This randomized clinical study involved 50 patients with chronic apical periodontitis and previous root canal treatment (ClinicalTrials.gov ID: NCT04072926). The inclusion criteria were previous endodontic treatment, asymptomatic inadequate endodontic treatment, PLs measuring > 5 mm, and percussion and palpation sensitivity. The exclusion criteria were immunocompromised status, pregnancy, periodontally compromised teeth, vertical root fracture, and antibiotic usage in the last month. Single-visit root canal retreatment was performed by the same endodontist. The patients were randomly divided into two groups based on the root canal sealer used: CSBS (BioRoot RCS) and epoxy resin-based sealer (ERBS) (AH Plus). Periapical healing, as the primary outcome measure, was determined according to the reduction in PL volume on CBCT from the preoperative period to the 1-year postoperative period. **Results:** Pre- and postoperative PL volumes (*p* > 0.05) were not significantly different between the CSBS and ERBS groups. The success rate (loose criteria) was 82.9% in the ERBS group and 94.7% in CSBS group. **Conclusions:** Root canal retreatment outcomes of CSBS, including periapical healing, are comparable to those of ERBS at 1 year after the retreatment.

## 1. Introduction

Calcium silicate-based sealers (CSBSs), also known as bioceramic sealers, have become popular in endodontic treatment during the last decade owing to their superior biological and mechanical properties: biocompatibility, bioactivity, antimicrobial activity, and dimensional stability [1,2,3,4]. CSBSs are also called hydraulics because of their reaction with water during setting, during which high amounts of calcium hydroxide are released [2]. This causes the local pH to increase to 12, resulting in significant antibacterial activity [5,6]. The released calcium hydroxide interacts with the surrounding phosphate, creating a hydroxyapatite precipitate [2]. This is particularly significant in the contact area of the sealer with dentin [1], where tight micromechanical and chemical bonds are created [7,8]. Moreover, CSBSs can be set in the surrounding moisture, a significant clinical benefit [2].

Another clinical benefit of CSBSs is their dimensional stability without shrinkage, which enables their application in larger volumes in the root canal [1]. CSBSs are considered a cement and recommended for use as the main component of root canal fillings with a single-cone (SC) obturation technique [8]. This large-volume application addresses the current root canal filling issue of applying a minimal amount of sealer owing to its shortcomings [9]. Furthermore, the SC obturation technique is simple and cost effective, making it the most commonly used obturation method (63.3%) among dentists and general practitioners using CSBS [10]. In the SC technique with CSBS, the gutta-percha cone is intended to deliver the sealer by hydraulic pressure inside the root canal and into intracanal irregularities and to act as a pathway for retreatment and post-space preparation [11]. This benefit of CSBS can be significant in clinical practice because it enables the use of a simple obturation technique that can be performed by both specialist endodontists and general practitioners.

The physicochemical and biological properties of CSBSs have been evaluated and compared to those of traditional sealers in many in vitro studies [12,13,14]. CSBSs have been demonstrated to reduce the periapical inflammatory response and contribute to the healing of PL owing to their bioactivity [15,16]. However, few clinical studies have evaluated the radiographic and clinical outcomes of root canal treatments after CSBS [17]. Although some studies evaluated the incidence of postoperative pain, the results were conflicting [18,19]. A recent meta-analysis [18] concluded comparable tooth survival, treatment outcomes, postoperative pain, and extruded material between premixed CSBS and standard sealers. Additionally, although the setting time of CSBS begins upon contact with moisture, the most suitable level of moisture in the root canal has not yet been reported, which may compromise clinical use [18]. Moreover, although the biological properties of CSBSs have been well-researched, their clinical behavior and possible application in SC obturation are still not well understood.

Hence, this study aimed to examine the 1-year healing rate of PL after single-visit root canal retreatment and root canal filling using a CSBS with SC obturation technique. To this end, we compared the outcomes with those of the epoxy resin-based sealer (ERBS) in combination with cold lateral condensation (CBC). The null hypothesis was that there would be no significant difference in the radiographic outcomes between CSBS and ERBS in single-visit root canal retreatment.

## 2. Materials and Methods

### 2.1. Study Design and Ethics

**Figure 1:**
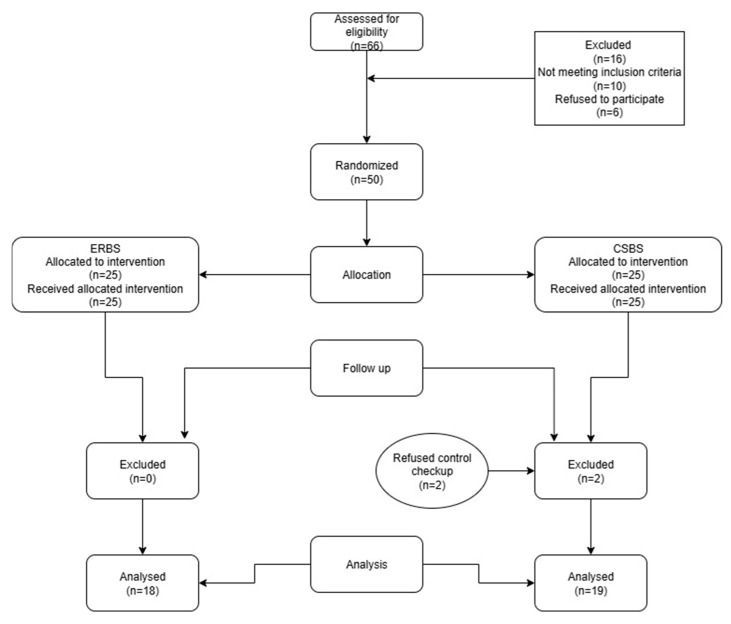
Flow diagram of the study. ERBS, epoxy resin-based sealer. CSBS, calcium silicate-based sealer.

This single-blind randomized clinical study was conducted at a public dental polyclinic between June 2019 and December 2023. This study was registered at clinicaltrials.gov (id 021/002-19-208 NCT04072926). The Local Ethics Committee confirmed the study protocol 12.04.2019. (number N0 05-PA 30-VI-/2019). The whole study protocol was performed in accordance with the Declaration of Helsinki. This study followed the published guidelines for randomized trials CONSORT 2025 [20] and the flow diagram is presented in Figure 1.

The sample size was calculated statistically using analysis of variance based on two groups and three repeated measures. The level of significance was set at α = 0.05, with a statistical power of 90%. Randomization was conducted using the Wheel Decision Program (www.wheeldecide.com). The person who did the randomization was not included in the further research protocol and evaluation. As the study was single blind, the patients were unaware of the assigned groups. The clinical procedures were standardized for both groups. The study was registered at clinicaltrials.gov id 021/002-19-208 NCT04072926 (27 August 2019).

### 2.2. Patients

Patients who had been referred to the Department of Endodontics at the Dental Polyclinic for root canal retreatment were included. The inclusion criteria were as follows: (1) asymptomatic inadequate endodontic treatment, (2) teeth sensitive to percussion and palpation, and (3) apical periodontitis (AP) > 5 mm in diameter based on the initial periapical radiograph. The exclusion criteria were as follows: (1) teeth with spontaneous pain and swelling, (2) signs and symptoms of vertical root fracture, (3) severely damaged teeth with no opportunity for prosthetic or restorative rehabilitation, (4) antibiotic intake in the last month, (5) immunocompromised status, (6) pregnancy, and (7) teeth with pocket depth > 3 mm [21]. All patients were examined clinically, and the teeth were analyzed using periapical radiography. Before inclusion in the study protocol, the selected patients had to sign an informed consent confirming their free will to participate in the study. Also, they were able to stop participating in the study at any time during the research.

In total, 50 patients (27 men and 23 women) aged 20–55 years were initially included (Figure 1). After initial examination, they were referred to undergo cone beam computed tomography (CBCT) (Cranex 3DX, Soredex, Tuusula, Finland) of the selected tooth to measure the baseline preoperative periapical lesion (PL) volume.

### 2.3. Root Canal Chemomechanical Treatment

All patients underwent single-visit root canal retreatment by the same practitioner (BP), who was a specialist in endodontics with more than 15 years’ experience. The retreatment protocol was standardized for all patients. The patients were randomly distributed into two experimental groups depending on the filling material used:

Group 1. Calcium silicate-based sealer, BioRoot RCS (Septodont, Saint-Maur-des-Fossés, France)

Group 2. Epoxy resin-based sealer, AH Plus (Dentsply Sirona, Bensheim, Germany).

The clinical procedures of retreatment were performed under local plexus anesthesia (4% with epinephrine 1:100,000, 1 ampoule, UbistesinTM Forte, 3M ESPE, Bavaria, Germany). Then, the rubber dam was placed on the tooth that required retreatment. The traditional access opening was performed using a diamond round and fissure bur under water cooling, and the caries dentin was removed with round carbide burs. In patients with a significant loss of coronal tooth structure, class I cavities were reconstructed using an adhesive technique (G-Premio Bond, GC, Tokyo, Japan) and composite material (G-aenial, GC, Tokyo, Japan). After the placement of the rubber dam, the whole working field was disinfected with 5% NaOCl.

Root canal retreatment was performed using R-endo Retreatment files 1 (25.08), 2 (25.06), and 3 (25.04) rotary files (Micro-Mega, Cedex, Besançon, France) in combination with 3% NaOCl used with a 30G needle (Steri Irrigation Tips, DiaDent; Almere, The Netherlands). The retreatment files were used with an endomotor (Reciproc Silver, Dentsply Sirona, Charlotte, NC, USA) with parameters set at 300 rpm and 200 Ncm, according to the manufacturer’s recommendations. The retreatment was considered complete when no signs of remaining gutta-percha were visible on the instruments or in the canal observed under a dental loupe with a magnification of 4.5 (Orascoptic, Madison, WI, USA). Then, the working length (WL) was determined using a K-file size of #10 or #15 up to the external apical foramen, which was confirmed using a Dualpex apex locator (Micro-Mega) up to level 0.0. Following the WL establishment, the canals were instrumented up to the external apical foramen using 2Shape 1 (25.04) and 2Shape 2 (25.06) instruments (2Shape; Micro-Mega) and irrigated with 5 mL 3% NaOCl per canal. Each set of instruments was used for only one patient. Adequate root canal cleaning was defined as the presence of clean dentinal dust, clear irrigants, and smooth glassy walls. Apical cleaning was confirmed when clean dentinal debris was present at the tip of the rotary instrument.

After completion of chemomechanical instrumentation, the final disinfection protocol was applied. It included root canal irrigation, first with 3 mL 3% NaOCl for 30 s. After 30 s, the remaining NaOCl was aspirated from the root canal using a 30G needle and 2 mL syringe, and the canal was then irrigated with 3 mL 15% ethylenediaminetetraacetic acid (EDTA) for 60 s. After removal of the remaining EDTA using the needle and syringe, the canals were finally irrigated with 3 mL of 3% NaOCl for 30 s [22]. The irrigation procedure was performed using a 30G needle (Steri Irrigation Tips, DiaDent, The Netherlands) and a 2 mL syringe. The irrigants in the final disinfection protocol were constantly activated using an EndoUltra device 3 (Micro-Mega) with an ultrasonic non-cutting tip placed 3 mm from the WL (ultrasonically activated irrigation, UAI). The irrigant was delivered into the root canal, and the ultrasonic device was constantly activated for 30 s for NaOCl and 60 s for EDTA. During activation, the irrigants were constantly delivered using the 30 G needle and syringe. The root canals were then dried using paper points.

### 2.4. Root Canal Obturation Protocol

Following the final disinfection protocol, the root canals were dried using sterile paper points (Micro-Mega). For the obturation, gutta-percha cones (MICRO-MEGA) and the sealers were used; in the CSBS group, the canals were obturated using single-cone technique (SC), and, in the ERBS group, cold lateral condensation technique (CLC) was used. First, the master point gutta-percha was chosen according to the final instrument used, and we checked whether it fit adequately to the WL based on tug-back. The root canal sealers were prepared according to the manufacturer’s instructions. The sealer was inserted into the root canal with a gutta-percha point and gently adjusted to the WL while spreading onto the dentinal walls. After the gutta-percha point was set to the WL, canal filling was performed. In the CSBS group, the gutta-percha access was cut with a hot plugger, and canal filling was vertically condensed with a cold plugger (Buchanan hand plugger, Kerr Dental, Brea, CA, USA). In the ERBS group, additional gutta-percha points sized #20 or #25 were inserted into the canal after lateral condensation using a manual spreader sized #20 or #20 (Dentsply Sirona).

After the canal was completely obturated, meaning that no extra accessory gutta-percha points could be introduced, the access gutta-percha was cut up to the canal entrance, and a manual plugger (Buchanan plugger, Kerr) was applied for vertical compaction. The access cavity was temporarily sealed with a glass ionomer cement (Fuji IX; GC Corporation, Tokyo, Japan). A control radiograph of the tooth was obtained to check the quality of the filling.

### 2.5. Cone Beam Computed Tomography Evaluation

The practitioner (BP) who performed the clinical procedures was not involved in the CBCT analysis. All patients underwent CBCT to measure PL volume before the procedure (initial CBCT) and 1-year post-root canal filling (follow-up CBCT). All scans were performed using the same device with standardized parameters (endo mode, 5 × 5 field of view, 0.085 mm voxel size, 450 mGY/mm^3^, 6.3 mA, 90 kV, 8.7 s) for analysis of the endodontic space and evaluation of PLs. CBCT were evaluated, and the volumes of PLs were calculated by two independent researchers, who were not involved in the study. If the volume of the measured lesions differed between researchers, the mean value was recorded. All CBCT analyses were performed using the same computer.

**Figure 2:**
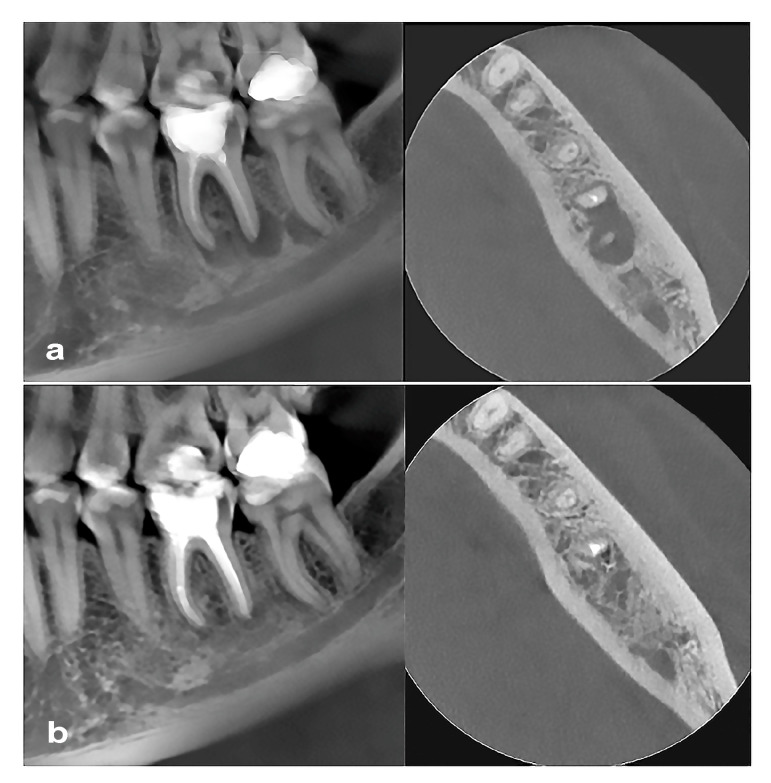
Representative CBCT of a lower third molar with inadequate root canal filling and large periapical lesion (**a**), and at 1-year follow-up (**b**) showing significant healing of the lesion after root canal filling with BioRoot RCS.

The initial CBCT and the control CBCT (1-year follow-up) were analyzed at intervals of >1 week. The volume of the PLs was measured using the 3D mode of the On-Demand software (OnDemand3Dapp 1.0.10.7510) with semiautomatic segmentation and automatic measurement of the gray intensity of the lesion [21]. The highest resolution with a slice thickness and interval of 0.125 mm was used for the detection of area segmentation and volume calculations. Periradicular defects were selected using a grayscale value range selection tool. If multirooted teeth had more than one PL, the individual lesions’ volumes were calculated together [23,24]. Follow-up measurements were performed 1 year after root canal retreatment. Assessment of the radiological volume of PL healing was performed using CBCT analysis by measuring the exact PL volume preoperatively and one year after the retreatment (control scan) (Figure 2). Healing of periapical lesion was defined as reduction in or absence of periapical radiolucency. Periapical radiolucency with volume less than twice the width of the periodontal ligament was counted as a 0 mm^3^ defect size [23,24]. Individual defect volumes in multirooted teeth were added together [23,24].

### 2.6. Statistical Analysis

Statistical analyses were performed using the chi-square test, Fisher’s exact test, and the Fisher Freeman–Halton test, with statistical significance set at 5%. For the statistical analysis, SPSS Statistics version 25 (IBM Corp., Armonk, NY, USA) was used.

## 3. Results

The recall rate was 96%, with 48 patients evaluated 1 year posttreatment. Two patients from the CSBS group refused control check-up. There were no significant between-group differences with respect to age (*p* = 0.475) or initial PL volume (*p* = 0.395). In the ERBS group, 23.5% of teeth were incisors; 17.64%, premolars; and 58.82%, molars. In the CSBS group, 10.52% of teeth were incisors; 31.58%, premolars; and 57.89%, molars.

Importantly, PL volume at the 1-year follow-up (*p* = 0.422) and the reduction rate in PL volume at 1 year posttreatment (*p* = 0.458) was not significantly different between the two groups (Table 1). The failure rates (only enlargement of initial PL recorded) were 10.81% in the total population, 16.7% in the ERBS group, and 5.3% in the CSBS group. The success rates (loose criteria) were 89.19% in the total population, 82.9% in the ERBS group, and 94.7% in the CSBS group. Complete healing was achieved in 44% and 21% of the patients in the ERBS and CSBS groups, respectively.

## 4. Discussion

The current study shows that CSBS (BioRoot RCS) used with the SC obturation technique during a single-visit retreatment provides a similar PL healing rate as ERBS (AH Plus). These results confirm our null hypothesis that CSBS can be an alternative to ERBS for root canal retreatment.

The increased popularity of calcium silicate-based materials in endodontics and their wide manufacturing over the past decade have led to many published studies on their properties and behavior in the root canal [2]. CSBS is superior to other root canal sealers with respect to biocompatibility, bioactivity, dimensional stability [2,4], calcium ion release, and increased pH [2,14,25]. Therefore, there is a new recommendation for the use of CSBS with SC obturation, which is simpler and more accessible to general dentists. However, there are some clinical concerns, including periapical healing, postoperative pain, extrusion rate, and the fate of the extruded material [18].

Our findings about BioRoot/SC obturation are similar to those reported by Bardini et al. [26], who found a lower 12-month failure rate in the BioRoot/SC (CSBS) group than in the control group (zinc-oxide eugenol sealer with warm vertical compaction (WVC)) (2.6% vs. 6.7%). In the current study, which included patients with enlarged PL, the failure rates were 5.3% in the CSBS/SC group and 16.7% in the ERBS/CLC group. In a recent retrospective study by Li et al. [27], the overall failure rate in the premixed iRoot SP/SC technique was 4.2%. In another study by Bardini et al. with a 4-year follow-up [28], root canal treatment with CSBS (BioRoot RCS) using the SC obturation technique demonstrated an 89.6% success rate, and this was highly similar to that using zinc-oxide eugenol sealer/WVC. Similarly, Simone et al. [29] found a high success rate of BioRoot/SC (87.7%) at the 24-month follow-up. In the current study, the success rate in both groups was also high at 94.7% in the CSBS group and 82.9% in the ERBS group.

Only few studies on the clinical outcomes of CSBS in retreatment cases have been published [17]. Sabeti et al. [17] recently reviewed five clinical studies and found a retreatment failure rate of 5.2% after new canal filling with different CSBSs using the SC technique. They concluded that CSBS in combination with the SC obturation technique provided clinical outcomes similar to those of other obturation techniques and materials (WVC, continuous wave of condensation with AH Plus or ZNE). A closely similar failure rate was found in the current study (5.3%). Kim et al. [30] also reported a comparable failure rate of primary root canal treatment and retreatment between CBSC (Endoseal) in combination with the SC obturation technique and AH Plus sealer in combination with CWC (5.7% vs. 7.7%) after a 17-month follow-up.

In the current study, the PL reduction rate was similar between the two groups: 86.95% in the CSBS group and 86.98% in the ERBS group. The similar and high success rates in both groups may be attributed to the disinfection protocols used. In both groups, the irrigants (NaOCl, EDTA, and NaOCl) in the final irrigation protocol were activated using ultrasonically activated irrigation, possibly resulting in a high bacterial eradication rate [22,31]. However, this remains to be confirmed as some recent randomized clinical studies did not prove the influence of the irrigation technique on PL healing [32]. The current study evaluated the outcomes of single-visit root canal retreatment after 1 year, which may be a short evaluation period. However, short-term studies have clinical significance with respect to reporting treatment failures; therefore, we also discussed treatment failures and compared them with those in other studies. In contrast, longer follow-up periods are clinically significant in reporting treatment success [33]. In a very recent study [34], a very high 3-years survival rate of 92.7% and the success rate of 85.4% was reported for Ceraseal premixed CSBS with single-cone technique, evaluated by periapical index score. In this study, the success rate of ERBS and CSBS (BioRoot RCS) used with the single-cone technique was above 80% after one year showing that the 1-year follow-up is a good predictor of successful healing outcome.

Most published studies [17,34] evaluated treatment outcomes using periapical radiographs and measured the periapical index. Meanwhile, the present study assessed retreatment outcomes, including healing, using CBCT. CBCT is a more sensitive and, therefore, more accurate method for the evaluation of the prognosis of root canal treatment [35]. It is also a recommended method for evaluating the clinical outcomes of different endodontic protocols [36]. Many previous studies have shown that the success rate of root canal treatment assessed using CBCT is lower than that determined using periapical radiography.

This study has limitations. First, the study included different types of teeth (incisors, premolars, and molars), which could have influenced the treatment outcome. The success rate of endodontic treatment depends on the tooth type, with success rates being lower in molars with more complex canal anatomy than in single canal teeth [37]. However, an advanced ultrasonically activated irrigation technique was used for all samples, providing successful cleaning and disinfection. Furthermore, the study only included 48 patients. The sample size may have been too small to demonstrate a difference between the two protocols. However, the study did not aim to compare the two protocols but rather to test the efficacy of CSBS (BioRoot RCS) in combination with the SC technique.

## 5. Conclusions

CSBS with the SC obturation technique has similar success and failure rates to ERBS in combination with CLC and can thus be a possible alternative, as it is widely accessible to general dentists.

## Figures and Tables

**Table 1:** Volume of periapical lesion (mm^2^) at baseline and after 1-year follow-up, and the reduction in periapical lesion in BioRoot RCS and AH Plus group.

Volume of Periapical Lesion	Experimental Groups	Minimal	Maximal	Percentiles		
				25th	Median	75th
Baseline	AH Plus	117.71	15,549.9	188.92	330.16	975.73
	BioRoot RCS	95.44	12,663.9	173.73	533.77	1284.84
At 1 year	AH Plus	0	8575.65	0	30.57	355.34
	BioRoot RCS	0	1068.61	49.52	110.78	437.44
Reduction in periapical lesion volume after 1 year (%)	AH Plus	11.13	100.00	37.23	86.98	100.00
	BioRoot RCS	6.64	100.00	34.77	86.95	91.76

## Data Availability

Data is unavailable due to privacy or ethical restrictions.

## Abbreviations

The following abbreviations are used in this manuscript:

AP	Apical periodontitis
CBCT	Cone beam computed tomography
CLC	Cold lateral condensation
CSBSs	Calcium silicate-based sealers
EDTA	Ethylenediaminetetraacetic acid
ERBS	Epoxy resin-based sealer
PL	Periapical lesion
SC	Single cone
UAI	Ultrasonically activated irrigation
WL	Working length
WVC	Warm vertical compaction
